# Frontal White Matter Hyperintensities Effect on Default Mode Network Connectivity in Acute Mild Traumatic Brain Injury

**DOI:** 10.3389/fnagi.2021.793491

**Published:** 2022-02-16

**Authors:** Danbin Zhang, Pingyi Zhu, Bo Yin, Pinghui Zhao, Shan Wang, Limei Ye, Lijun Bai, Zhihan Yan, Guanghui Bai

**Affiliations:** ^1^Department of Radiology, The Second Affiliated Hospital and Yuying Children’s Hospital, Wenzhou Medical University, Wenzhou, China; ^2^Department of Radiology, The First Affiliated Hospital, Zhejiang University School of Medicine, Hangzhou, China; ^3^Department of Neurosurgery, The Second Affiliated Hospital and Yuying Children’s Hospital, Wenzhou Medical University, Wenzhou, China; ^4^The Key Laboratory of Biomedical Information Engineering, Ministry of Education, Department of Biomedical Engineering, School of Life Sciences and Technology, Xi’an Jiaotong University, Xi’an, China; ^5^Department of Radiology, Jinhua Municipal Central Hospital and Jinhua Hospital of Zhejiang University, Jinhua, China; ^6^Wenzhou Key Laboratory of Basic Science and Translational Research of Radiation Oncology, Wenzhou, China

**Keywords:** default-mode network, fMRI, functional connectivity, traumatic brain injury, white matter hyperintensity

## Abstract

The functional connectivity of the brain depends not only on the structural integrity of the cortex but also on the white matter pathways between cortical areas. White matter hyperintensities (WMH), caused by chronic hypoperfusion in the white matter, play a role in the outcome of traumatic brain injury (TBI) and other neurodegenerative disorders. Herein, we investigate how the location and volume of WMH affect the default-mode network (DMN) connectivity in acute mild TBI (mTBI) patients. Forty-six patients with acute mTBI and 46 matched healthy controls were enrolled in the study. All participants underwent T2-weighted fluid-attenuated inversion recovery magnetic resonance imaging (MRI), resting-state functional MRI (fMRI),and neuropsychological assessments. The volume and location of WMH were recorded. The relationships between the WMH volume and clinical assessments were evaluated using Spearman’s correlation. Patients with higher frontal lobe WMH volume had more severe post-concussion symptoms and poorer information processing speed. Moreover, these patients had significantly lower functional connectivity in the right middle temporal gyrus, left middle frontal gyrus, right superior frontal gyrus, and left anterior cingulate cortex, compared with patients with low frontal lobe WMH volume. Compared to the controls, the patients with high frontal WMH volume exhibited significantly lower functional connectivity in the right inferior temporal gyrus, left anterior cingulate cortex, and right superior frontal gyrus. These findings suggest that frontal lobe WMH volume may modulate the functional connectivity within the DMN. Therefore, the WMH volume in specific regions of the brain, particularly the frontal and parietal lobes, may accelerate the process of aging and cognitive impairment may be a useful biomarker for the diagnosis and prognosis of acute mTBI.

## Introduction

Traumatic brain injuries (TBI) is a public health challenge of vast, but insufficiently recognized, proportions, of which70–90% of TBIs are classified as mild traumatic brain injury (mTBI) (Holm et al., 2005). The mTBI patients frequently report neurologic and psychologic complaints, which usually appear within weeks and may persist for months to years (Barry Willer, 2006). Some authors have suggested that mTBI is a process of neuronal plasticity that leads to long-term structural and functional alterations to the brain (Dall’Acqua et al., 2017). However, these clinical symptoms are not generally associated with visible abnormalities on routine magnetic resonance imaging (MRI) or computed tomography (CT) (Raikes et al., 2018). More recently, advanced techniques such as diffusion tensor imaging (DTI) and functional magnetic resonance imaging (fMRI) have been frequently used to assess both the functional and structural connectivity alternations following mTBI (Cubon et al., 2011; Vakhtin et al., 2013; Salat et al., 2017).

Resting-state fMRI (rs-fMRI) can be used to evaluate the functional brain networks of mTBI patients with cognitive deficits. The default mode network (DMN) is part of the most studied “resting-state” brain networks (Fox and Raichle, 2007), which are activated during the rest and suppressed during the execution of attention and purposeful tasks (Zhang and Raichle, 2010). The posterior cingulate cortex (PCC), precuneus, inferior parietal, and medial prefrontal cortex (MPFC) are comprised of the typical DMN (Raichle and Snyder, 2007). The mTBI patients have been consistently exhibited with abnormal functional connectivity within the DMN (Mayer et al., 2011; Zhou et al., 2012). Furthermore, the alternated functional connectivity was also related to worse neurocognitive performance, which can be served as a biomarker to track disease progression and recovery in mTBI (Zhou et al., 2012).

The functional connectivity of the brain also depends on the white matter pathways connecting the cortical areas. Therefore, intact white matter connections in the brain are important for the processing and integration of information generated by neural networks. White matter hyperintensities (WMH), as one of the macroscopic process affecting the white matter, is commonly seen in aging and which are readily recognized in both radiologic and pathologic examinations (de Leeuw et al., 2001). One recent post-mortem imaging and histology evidence demonstrates that mTBI induces WMH co-localization with iron-laden macrophages, suggesting a cerebrovascular origin (Griffin et al., 2019). Meanwhile, recent studies have suggested that WMH may affect cognitive functions through hemispheric gray matter atrophy/lateralization (Gan et al., 2021), the similar influence might be applied to mTBI.(Biesbroek et al., 2013; Gan et al., 2021). However, the contribution of WMH to the impairment of functional networks underlying cognitive changes is not well-understood in mTBI. A recent rs-fMRI study showed that a higher WMH burden is associated with impaired brain function (Zhou et al., 2015). However, these studies used global measures of WMH volume averaged across the entire white matter (DeCarli et al., 1995). Recent studies on ischemic WMH have highlighted the relevance of lesion location and anatomical connections between WMH and cortical gray matter (Biesbroek et al., 2013). The IFOF connecting between DMN brain regions is also reported to be loss of integrity following mild TBI in our previous study (Yin et al., 2019). Applied to mTBI, we hypothesized that higher WMH volume in anatomically defined white matter is associated with functional brain changes specifically in connected cortical regions. We selected DMN, which is most likely to be injured and influence metacognitive accuracy, cause cognitive decline in mTBI, as the target network.

To test this hypothesis, we measured the effect of WMH on functional connectivity (FC) within the DMN, which can be readily identified during rs-fMRI in patients with mTBI. We focused on FC changes within the DMN because this is the major functional neural network that shows reduced FC in mTBI and for which the white matter connections between different DMN regions have been demonstrated (Fernandez-Espejo et al., 2012). Specifically, we tested whether frontal WMH is associated with reduced FC in the DMN.

## Materials and Methods

### Participants

We prospectively recruited 46 patients with acute mTBI (≤7 days post-injury) who presented to the Emergency Department of a local hospital between August 2016 and July 2017. All mTBI participants’ medical records were checked to confirm that they were initially visited patients in the present study, and they had also met the criteria of the World Health Organization’s Collaborating Centre for Neurotrauma Task Force (Holm et al., 2005). (i) a score of Glasgow Coma Scale (GCS) 13–15 on patients to the Emergency Department; (ii) at least one of the following characteristics: loss of consciousness <30 min, post-traumatic amnesia (if present) <24 h, and/or other transient neurological abnormalities such as focal signs, seizure, and an intracranial lesion that do not require surgery; (iii) age ranging between 18 and 60, without symptomatic treatment drugs, diagnosed within 1 week of having experienced mTBI.

Exclusion criteria for mTBI patients were as follows: (i) a fact of a prior brain injury, neurological conditions, long-standing psychiatric condition, or concurrent substance or alcohol abuse; (ii) moderate or severe TBI (i.e., loss of consciousness >30 min, alteration of consciousness or post-traumatic amnesia > 24 h or Glasgow Coma Scale > 15; (iii) a structural abnormality on neuroimaging (CT and MRI); (iv) the manifestation of mild TBI due to medications by other injuries (e.g., systemic injuries, facial injuries, or spinal cord injury); (v) other problems (e.g., a skull fracture and administration of sedatives psychological trauma, language barrier, or serious medical illness); (vi) any contraindications that would exclude MRI. Based on these conditions, 46 patients (23 males) were enrolled. In addition, 46 sex-, age-, and education-matched healthy controls (HCs; 21 males) without neurologic impairment or psychiatric disorders participated in the study. Participants were all right-handed according to the Edinburgh Handedness Inventory (Espirito-Santo et al., 2017).

All participants provided written, informed consent in person. This study was approved by the Ethical Committee of The Second Affiliated Hospital of Wenzhou Medical University and conducted in accordance with the tenets of the Declaration of Helsinki.

### Clinical Assessment

All the participants were performed for Clinical assessments within 48 h of MR imaging. Neuropsychological tests were used in this study: (i) WAIS-III Digit Symbol Coding (DSC) and Trail-Making Test Part A to examine the speed of processing cognitive information; (ii) Forward Digit Span (FDS) and Backward Digit Span from the WAIS-III, to assess working memory (Harman-Smith et al., 2013); (iii) Verbal Fluency Test to assess verbal fluency including language ability, semantic memory, and executive function (Joy et al., 2004); (vi) Beck Depression Inventory-II (BDI-II) to examine depression severity (Lehmicke and Hicks, 1995); (v) PTSD Checklist—Civilian Version (PCL-C) (Ruggiero et al., 2003); (vi) Fatigue Severity Scale (Krupp et al., 1989)and Insomnia Severity Index (ISI) (Sadeghniiat-Haghighi et al., 2014); (vii)Post-concussive symptoms (PCS) measured with the Rivermead Post-Concussion Symptom Questionnaire.

### Image Acquisition

A non-contrast CT scan was performed on all acute head injury patients with a 64-row CT scanner (GE, Lightspeed VCT). MRI was performed using a 3.0 T GE Discovery MR750 scanner with a 32-channel receive-only head coil.

We used a custom-built head holder to prevent head movements. Participants were instructed to lay supine and avoid engaging in any mental activities for the entire duration of the MRI protocol. Alertness during the scan was confirmed when at scan completion. These MRI sequences were used in this study: a high-resolution three-dimensional T1 (3DT1) sequence [echo time (TE) = 3.17 ms, repetition time (TR) = 8.15 ms, flip angle (FA) = 9^°^, slice thickness = 1 mm, field of view (FOV) = 256 mm × 256 mm, matrix size = 256 × 256], single-shot, gradient-recalled echo planar imaging (EPI) sequence with 54 slices covering the whole brain (TR = 2,500 ms, TE = 30 ms, slice thickness = 3 mm, FA = 90^°^, FOV = 216 mm × 216 mm, matrix size = 64 × 64, voxel size = 3 mm × 3 mm × 3 mm), a axial three dimensional T2-weighted fluid-attenuated inversion recovery (FLAIR; TR = 9,000 ms, TE = 95 ms, FA = 150^°^, thickness = 5 mm, slices = 20, FOV = 240 mm × 240 mm, matrix size = 173 × 256). DTI scan (b = 1,000 s/mm^2^) were acquired with 30 diffusion gradient orientations and the b = 0 repeated two times (acquisition time = 9:28 min) and 3D ASL including M0 image and perfusion different image with the parameters [TR = 5,046 ms, TE = 11 ms, slice thickness = 3 mm, field of view (FOV) = 24 × 24 mm, labeling time = 1.5 s, post-labeling delay = 2,000 ms, acquisition time = 4:53 min].

The presence of focal lesions and cerebral microbleeds was independently determined by two experienced clinical neuroradiologists (each with 10 years’ experience) who assessed the multiple modalities of neuroimaging data acquired [T1-weighted (T1-w), susceptibility-weighted, fluid-attenuated inversion recovery (FLAIR)]. Any disagreement between these two observers was resolved by consensus. The examiners were blinded to clinical information and group membership (patient or control).

### White Matter Hyperintensities Lesion Distribution Analysis

We initially assessed WMH volumes in four cerebral lobes and whole brain WMH volumes respectively. According to Valverde et al. (2015), it is necessary to refill white matter lesions before tissue segmentation for accurate cross-sectional tissue volume measurements. Automated lesion segmentation and filling were performed using the T1-w and FLAIR image modalities on publicly available toolkits implemented for the SPM software package: The Lesion Segmentation Tool (LST). The LST pipeline was composed of the following automated steps: 3DT1 and T2-FLAIR images of each subject were used to automatically segment WMH lesions using the lesion growth algorithm (LGA) in SPM12.^1^ Next, all voxels in the 3DT1 images were labeled as gray matter (GM), white matter (WM), or cerebrospinal fluid. The hyper-intense regions of each tissue class were extracted based on the T2-FLAIR images.

Then, corrected T1-w and FLAIR images were linearly (12-parameter affine) and non-linearly co-registered using the internal SPM12 routines. Lesion segmentation was performed by computing an initial tissue segmentation of the T1-w image to compute a lesion belief map based on the FLAIR and T1-w images (Schmidt et al., 2012). This map was refined iteratively, weighing the likelihood of belonging to WM against the likelihood of belonging to lesions until no further voxels were assigned to lesions. The required initial threshold kappa was set to k = 0.15, while the lesion belief map was set to lbm = GM. Estimated lesion masks were then automatically filled using an internal filling method inspired by a previous technique (Chard et al., 2010) in which candidate region voxels were replaced by random intensities from a Gaussian distribution generated from the normal-appearing WM intensities and then filtered to reintroduce the original spatial variation in WM.

We co-registered the standard atlas (UNC adult brain atlas template, created by the University of North Carolina at Chapel Hill)^2^ to the 3DT1 images of each subject. The UNC lobar parcellation mask had five different ROIs, encoding the frontal, occipital, temporal, parietal lobe, and subcortical regions. The 3DT1 image was co-registered to T2 FLAIR and the deformation field was applied to the registration of individual cerebral masks to T2 FLAIR images. These cerebral masks were used to extract the WMH volume in five cerebral ROIs by combing WMH lesion maps. We then sub-divided the mTBI patients into two groups according to whether their measured frontal lobe WMH volume was above (Patient-A) or below (Patient-B) the mean WMH volume in the frontal lobe in the healthy controls.

### Preprocessing of Resting-State Functional Magnetic Resonance Imaging Data

Resting-state fMRI preprocessing was performed using the Data Processing and Analysis of Brain Imaging (DPABI 2.3)^3^ software, which is based on statistical parametric mapping (SPM12). The first 10 volumes from each subject were discarded to account for the signal equilibrium and the subjects’ adaptation to the scanning noise. The remaining 152 volumes were corrected for acquisition-time differences between the slices and were subsequently realigned to the first volume. Afterward, the head motion parameters were calculated, and no participants’ head motion exceeded 2 mm in x, y, or z translation or 2° in rotation. The resulting functional data were spatially normalized to the Montreal Neurological Institute (MNI) space (using the mean EPI image as the source volume), resampled into 3 × 3 × 3 mm^3^ voxels and smoothed with a Gaussian kernel of 6 mm. The 24 head motion parameters, white matter signal, cerebrospinal fluid signal, and whole brain signal were regressed out.

### Definition of the Default-Mode Network Masks and Internetwork Functional Connectivity Analyses

We adopted a region of interest (ROI)-based method to extract the DMN. The definition of ROIs of the DMN was based on that in a previous task fMRI study (PCC as MNI coordinates: 0, −52, 27) (Duan et al., 2012). The time-courses of the mean BOLD signal of a 10-mm radius sphere centered at the peak coordinate of ROI was extracted to used as the model response function in the generalized linear model. For the first-level analysis, bivariate temporal correlations were conducted among individuals’ time series data from *a priori* ROI and all other voxels across the brain to produce PAG-seeded FC maps. Then, Fisher’s r-to-z transformation was applied to convert each individual-level correlation map into a z-FC map. The second-level analysis was performed to assess FC maps for group-level comparison. Voxel-wise one-way analysis of variance (ANOVA) was used to test for FC differences across three groups (Patient-A, Patient-B, and HCs) with age, gender, and education as nuisance covariate within a DMN mask. *Post hoc* analysis was also conducted for between-group comparisons (*P* < 0.01, FWE correction).

### Statistical Analysis

All statistical analyses were performed using SPSS (version 25, IBM Corp., New York, NY, United States) and Prism (Version 7, GraphPad Software, San Diego, CA, United States). The Kolmogorov-Smirnov test was used to test for normality in all continuous variables. The Mann-Whitney *U* test was used for variables that were not normally distributed. Normally distributed data were subjected to the independent two-sample *t*-test.

The Chi-squared test and non-parametric tests were utilized to compare the differences in demographic and neuroimaging characteristics, medical history, and neuropsychological data between the patients and controls, besides, the same procedures were used to compare between the Patient-A and Patient-B. The relationship between the volume of WMH and results of the clinical assessments were obtained using Spearman’s correlation. For all tests, a *P*-value <0.05 was considered significant.

## Results

### Demographic and Clinical Characteristics

Forty-six patients with mTBI (23 males, mean age 34.7 ± 13.2 years, mean education level of 9.1 ± 4.4 years) and 46 matched healthy controls (21 males, mean age of 35.9 ± 12.3 years, mean education level of 11.1 ± 5.9 years) were recruited for this study. No significant differences existed between the mTBI patients and healthy controls regarding age, education level, and gender (*P* > 0.05). The detailed demographic data and clinical characteristics of the participants are summarized in Table 1.

**TABLE 1:** Summary of demographic characteristic and neuropsychological tests cores between patients and controls at acute phase.

	mTBI patients	Controls	*P*-value
**Demographic**			
Age	34.7 ± 13.2	35.9 ± 12.3	0.494
Gender (M/F)	23/23	21/25	0.678
Education	9.1 ± 4.4	11.1 ± 5.9	0.053
**Neuropsychological test**			
TMT A	59.8 ± 44.9	39.5 ± 22.4	0.019
FDS	7.9 ± 1.7	8.3 ± 1.6	0.289
BDS	4.0 ± 1.6	4.5 ± 2.0	0.336
VF	17.4 ± 5.4	18.5 ± 6.1	0.362
DSC	38.3 ± 16.4	45.9 ± 17.2	0.016
**Self-report measures**			
PCS	10.8 ± 7.4	2.6 ± 3.0	< 0.001*
PCL-C	25.3 ± 6.6	17.0 ± 0.0	< 0.001*
FSS	10.4 ± 5.9	9.0 ± 0.0	0.080
BDI	4.6 ± 3.55	0.0 ± 0.2	< 0.001*
ISI	7.4 ± 6.4	1.8 ± 3.1	< 0.001*
**MTBI severity (N%)**			
GCS = 15	46 (100%)		
GCS = 13, 14	0 (0%)		

TMT A, Trail-Making Test Part A; FDS, Forward Digit Span Task; BDS, Backward Digit Span Task; VF, Verbal Fluency; DSC, Digit Symbol Coding; PCS, Post concussive Symptoms Scale; PCL-C, Post-Traumatic Stress Disorder Checklist Civilian; FSS, Fatigue Severity Scale; BDI, Beck Depression Inventory; ISI, Insomnia Severity Index; GCS, Glasgow Coma Scale. *P < 0.05.

Compared to healthy controls, mTBI patients exhibited lower performance in terms of information processing speed by the Digit Symbol Coding test (DSC; *P* = 0.016) and Trail-Making Test Part A (TMT A; *P* = 0.019). The mTBI patients also presented more complaints in the Post-Traumatic Stress Disorder Checklist (Civilian Version) (PSL-C) compared to the controls (*P* < 0.001). Moreover, patients reported significant discomfort in Post-Concussive symptoms (PCS; *P* < 0.001), also reported more symptoms of depression and insomnia in Beck’s Depression Inventory (BDI; *P* < 0.001), insomnia severity index (ISI) than those of the healthy controls (*P* < 0.001; Table 1).

### White Matter Hyperintensities Volume Subgroups

We calculated the WMH volume within the whole brain and four cerebral regions. The mean WMH volume in the frontal lobe in the healthy controls were 752.0 mm^3^. Patients with a WMH volume higher than 752.0 mm^3^ were categorized into the Patient-A group, and those with WMH volume lower than 752.0 mm^3^ were assigned to the Patient-B group. Group Patient-A consisted of 25 patients (15 male patients, mean age 36.8 ± 12.8 years, mean education level 8.6 ± 4.3 years), and Patient-B consisted of 21 patients (8 male patients, mean age 32.3 ± 13.6 years, mean education level 9.7 ± 4.6 years). No significant differences were found between these groups in terms of age, education level, and gender (*P* > 0.05). The detailed demographic data and clinical characteristics of these subgroups are summarized in Table 2.

**TABLE 2:** Summary of demographic characteristic and neuropsychological test scores between patients at acute phase.

	Patients-A	Patients-B	*P*-value
**Demographic**			
Age	36.8 ± 12.8	32.3 ± 13.6	0.143
Gender (M/F)	15/10	8/13	0.246
Education	8.6 ± 4.3	9.7 ± 4.6	0.33
**Neuropsychological test**			
TMT A	60.0 ± 50.2	59.6 ± 38.9	0.991
FDS	7.8 ± 1.5	8.2 ± 1.9	0.571
BDS	3.8 ± 1.2	4.3 ± 2.0	0.449
LF	17.1 ± 5.2	17.7 ± 5.8	0.808
DSC	39.4 ± 15.5	37.0 ± 17.7	0.604
**Self-report measures**			
PCS	10.2 ± 5.6	11.6 ± 9.1	0.956
PCL-C	25.1 ± 4.5	25.5 ± 8.7	0.611
FSS	10.2 ± 5.6	11.6 ± 9.1	0.053
BDI	4.3 ± 2.8	4.9 ± 4.3	0.807
ISI	7.4 ± 5.8	7.5 ± 7.1	0.649
**mTBI severity (N%)**			
GCS = 15	25 (100%)	21 (100%)	
GCS = 13, 14	0 (0%)	0 (0%)	

*TMT A, Trail-Making Test Part A; FDS, Forward Digit Span Task; BDS, Backward Digit Span Task; VF, Verbal Fluency; DSC, Digit Symbol Coding; PCS, Post concussive Symptoms Scale; PCL-C, Post-Traumatic Stress Disorder Checklist Civilian; FSS, Fatigue Severity Scale; BDI, Beck Depression Inventory; ISI, Insomnia Severity Index; GCS, Glasgow Coma Scale.*

**FIGURE 1:**
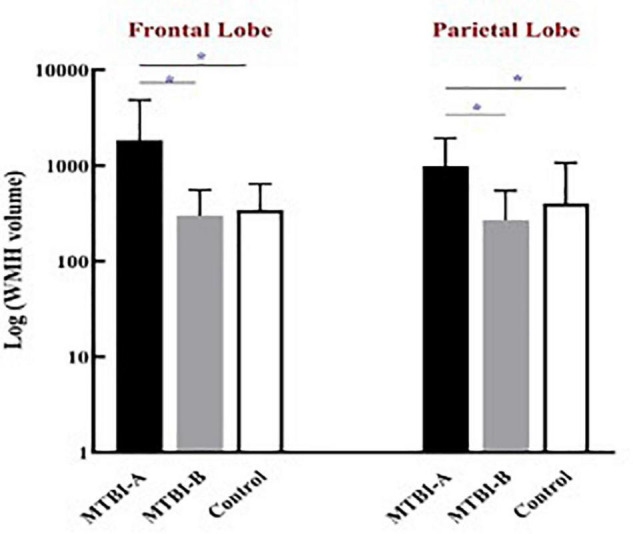
The frontal and the parietal WMH volume in Patient-A were significantly higher compared to both Patient-B (*P < 0.001) and controls (*P < 0.001).

The frontal WMH volume in Patient-A were significantly higher compared to both Patient-B (*P* < 0.001) and controls (*P* < 0.001). Meanwhile, the parietal WMH volume in Patient-A were significantly higher compared to both Patient-B (*P* < 0.001) and controls (*P* < 0.001) (Figure 1).

**FIGURE 2:**
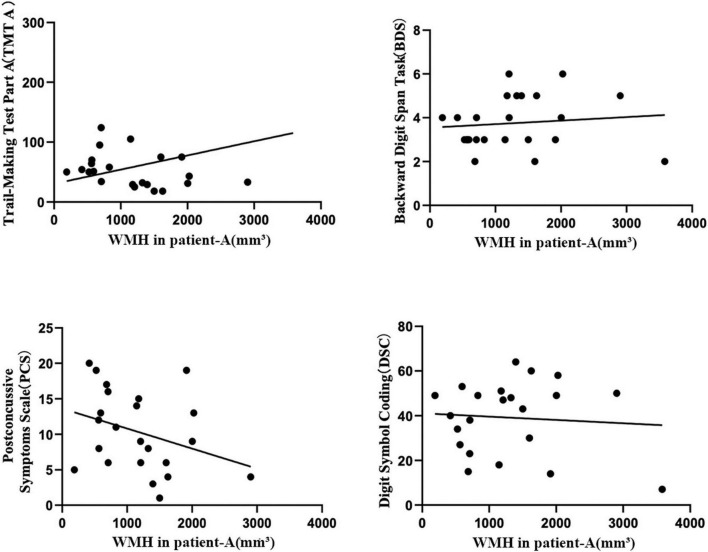
The Pearson correlation coefficient plots correlating the volume of frontal WMH in Patient-A with TMT A (*r* = 0.666, *P* = < 0.001*), BDS (*r* = –0.562, *P* = 0.003), PCS (*r* = 0.509, *P* = 0.009 C), and DSC (*r* = –0.590, *P* = 0.002) in the mTBI patients.

The volume of frontal WMH in Patient-A are associated with worse information processing speed outcomes from the TMT A (*r* = 0.666, *P* = < 0.001*), and DSC (*r* = −0.590, *P* = 0.002), a rapid decline in working memory form the BDS (*r* = –0.562, *P* = 0.003) and severer PCS (*r* = 0.509, *P* = 0.009) in the mTBI patients (Figure 2).

### Default Mode Network Results

We compared the FC within the DMN between the Patient-A and Patient-B groups, and found that patients with a high frontal lobe WMH volume had significantly decreased FC in the right middle temporal gyrus (rMTG), left middle frontal gyrus (lMFG), right superior frontal gyrus (rSFG), and left anterior cingulate cortex (lACC) compared with those in patients with a lower frontal lobe WMH volume (*P* < 0.01, FWE corrected).

**FIGURE 3:**
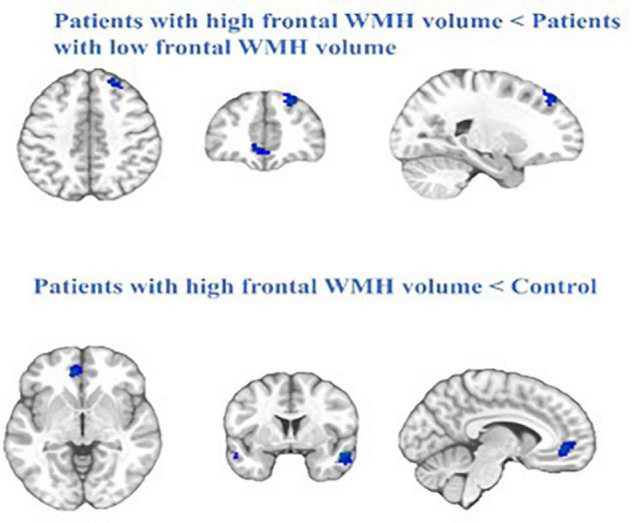
The blue represent regions of reduced RS-FC in the patients with high frontal WMH volume (Patient-A) compared with patients with low frontal WMH volume (Patient-B) and the control. Cluster extent threshold of *P*-value of 0.01 using a FWE correction for multiple comparisons.

When frontal lobe WMH volume was added as a regressor in the model, there was a negative effect on DMN connectivity maps in the mTBI group. Compared to the controls, the patients with high frontal WMH volume exhibited significantly lower functional connectivity in the right inferior temporal gyrus (rITG), lACC, and rSFG (*P* < 0.01, FWE corrected) (Figure 3).

## Discussion

Little is known about the relationship between WMH and FC in patients with TBI-related abnormalities. The present study confirms prior findings of reduced functional connectivity in disparate regions of the DMN network in mTBI patients and suggests a differential susceptibility of cerebral regions for vascular pathology according to the distribution of WMH. This is the first study to explore WMH as an observable biomarker of cerebral microvascular impairments and their effect on brain functional connectivity, which will aid both the diagnosis and prognosis of early acute-phase mTBI patients.

Compared with the healthy control group, the mTBI patients had lower scores for the DSC, and higher scores for the TMT-A, PCS, PCL-C, BDI, and ISI. The TMT-A is used to assess various aspects of cognitive function in both healthy subjects and patients, including processing speed, mental flexibility, visual-motor skills, and psychomotor processing speed in healthy subjects and patients (Ter Telgte et al., 2018). These results may explain the following neuropsychiatric symptoms in patients with mTBI: persistent headaches, lags in response, memory deficits, inattention, depression, emotional instability, and brief frustration after injury. WMH were relatively frequent among mTBI patients, and they induced a decrease in the healthy neural tissue available for the computations necessary to sustain cognitive functioning. The frontal and parietal WMH volume significantly increase in a portion of mTBI patients. The volume of frontal WMH is associated with severer outcomes from the TMTA, PCS, BDS, and DSC. Patients with high frontal WMH volume presented significantly lower functional connectivity in the rMTG, lMFG, rSFG, and lACG compared with patients with low frontal WMH volume. In addition, compared to the controls, the patients with high frontal WMH volume exhibited significantly lower functional connectivity in the rITG, lACG, and rSFG.

WMH is caused by traumatic axonal injury in the acute phase, then chronic hypoperfusion in the white matter, which results in demyelination as a consequence of repeated selective oligodendrocyte death (van Dalen et al., 2016). WMH increase with age and vascular disease, and is elevated in Alzheimer’s disease patients, suggesting that they might be good biomarkers of degeneration. Furthermore, WMH may accelerate the process of aging and cognitive impairment (Brickman et al., 2012; Birdsill et al., 2014; Cole and Franke, 2017). The accumulation of the WMH burden in frontal regions is closely tied to a decline in cognitive function (Gootjes et al., 2004; Tullberg et al., 2004). A major pathological basis of TBI is traumatic axonal injury, which is related to microhemorrhage and edema during the acute phase, and significantly reduces the functional connection of associative networks (Li et al., 2017). Experimental models of TBI have demonstrated that frontal and temporal brain regions are particularly vulnerable to impact during initial insult and memory deficits occur across various TBI samples (Clark et al., 2016). De Marco et al. (2017) suggested that the same cerebral territory appears to be affected by WMH load, including, structurally and functionally, the DMN. Our study found that there was no difference in the whole-brain WMH volume between the healthy controls and mTBI patients. However, a subset of the mTBI patients (Patient-A) had a significantly higher WMH volume in the parietal and frontal lobes, as compared to that in the controls. We hypothesized that during the acute phase of mTBI, the different localization patterns of WMH may reflect pathogenic differences. In other words, both the location and volume of WMH may affect the regional functional connectivity of gray matter, thus affecting cognition. In fact, we found that the volume of frontal lobe WMH is associated with worse outcomes on the TMTA, PCS, BDS, and DSC. The DMN, as a task-negative network involving self-reference processes, is involved with cortical networks governing cognition, alertness, perception, and memory (Buckner et al., 2008; Fingelkurts and Fingelkurts, 2011). Evaluation of the DMN facilitates the diagnosis of several cognitive pathologies such as attention deficit hyperactive disorder, schizophrenia, and Alzheimer’s disease (Whitfield-Gabrieli et al., 2009; Koch et al., 2012). Our results showed decreased functional connectivity between the rMTG, lMFG, rSFG, and lACC in group Patient-A, compared with that in group Patient-B. In addition, the patients in Patient-A exhibited significantly lower functional connectivity in the rITG, lACC, and rSFG, as compared to that in the controls. The brain regions involved in the DMN have emerged as a potentially novel and sensitive measure of subtle brain injury associated with diminished cognition and decision-making (He et al., 2012). The MTG and MFG are responsible for multimodal semantic cognition, verbal fluency, and language laterality (Jackson et al., 2016; Gohel et al., 2019), while the rSFG has been found to participate in inhibitory control (Hu et al., 2016), and the ACC has been associated with anxiety and post-traumatic stress disorder (Kennis et al., 2015).

Among healthy adults, the brain pursues adaptive plasticity by upregulating the connectivity of the DMN to compensate for the age-associated accumulation of white matter damage (De Marco et al., 2017). In this study, we specifically explored the negative associations between higher frontal lobe WMH volume and functional connectivity of the DMN to clarify which pathways of connectivity are downregulated when lesions accumulate in mTBI patients. Regarding the underlying mechanism, we speculate that projection fibers, which alter brain activity in vulnerable connected regions, may be directly damaged during the acute phase, resulting in functional disconnection. Thus, these factors should be considered when looking for relationships between subcortical structural abnormalities and connectivity. These findings suggest that a higher frontal lobe WMH volume can affect the connectivity of the DMN in patients with mTBI.

There were some limitations in the present study. First, we only explored changes in the DMN functional connectivity within the 1st week post-injury. The longer-term study is needed to elucidate the extent to which differences in WMH load can predict changes in brain structure and functional connectivity over time. Second, the sample size was relatively small. Third, we only considered resting-state fMRI; Using DTI to evaluate projection fibers integrity might reveal additional insights. Forth, the present study focused on the static functional connectivity, and growing evidence that brain networks are not immutable across time, but inherently “multistable.” It is suggested the subtler aspects of network disruption could become more apparent when considering dynamic functional connectivity patterns at the acute stage. Future research is needed to explore the relationship between structural and functional network defects and their long-term clinical significance.

## Conclusion

The mTBI may involve a progressive, neurodegenerative process, which resembles accelerated aging. Compared to healthy controls, only a subset of mBTI patients showed a significant increase in WMH volume, particularly in the parietal and frontal lobes. Significant negative associations were found between the higher frontal lobe WMH volume and functional connectivity of the DMN in acute mTBI patients. Our results suggest that the location and volume of WMH influence the effect of white matter damage on DMN connectivity and cognitive function. Therefore, WMH volume in specific brain regions may be a suitable biomarker for the prognosis of mTBI, and help facilitate early intervention to prevent adverse sequelae in such patients.

## Data Availability Statement

The raw data supporting the conclusions of this article will be made available by the authors, without undue reservation.

## Ethics Statement

The studies involving human participants were reviewed and approved by the Ethical Committee of the Second Affiliated Hospital of Wenzhou Medical University. The patients/participants provided their written informed consent to participate in this study.

## Author Contributions

DZ, LB, ZY, and GB contributed to the conception of the study. DZ contributed significantly to the analysis and manuscript preparation. SW, PyZ, and BY performed the data analyses and wrote the manuscript. GB, BY, PhZ, and LY helped perform the analysis with constructive discussions. All authors contributed to the article and approved the submitted version.

## Conflict of Interest

The authors declare that the research was conducted in the absence of any commercial or financial relationships that could be construed as a potential conflict of interest.

## Publisher’s Note

All claims expressed in this article are solely those of the authors and do not necessarily represent those of their affiliated organizations, or those of the publisher, the editors and the reviewers. Any product that may be evaluated in this article, or claim that may be made by its manufacturer, is not guaranteed or endorsed by the publisher.

## Footnotes

1
http://www.fil.ion.ucl.ac.uk/spm

2
www.nitrc.org

3
http://rfmri.org/DPABI
